# Development and Two-Year Follow-Up Evaluation of a Training Workshop for the Large Preventive Positive Psychology Happy Family Kitchen Project in Hong Kong

**DOI:** 10.1371/journal.pone.0147712

**Published:** 2016-01-25

**Authors:** Agnes Y. Lai, Moses W. Mui, Alice Wan, Sunita M. Stewart, Carol Yew, Tai-hing Lam, Sophia S. Chan

**Affiliations:** 1 School of Public Health, The University of Hong Kong, Hong Kong, SAR, China; 2 The Hong Kong Council of Social Service, Hong Kong, SAR, China; 3 Department of Psychiatry, University of Texas Southwestern Medical Center at Dallas, Dallas, Texas, United States of America; 4 United Centre of Emotional Health and Positive Living, United Christian Nethersole Community Health Service, Hong Kong, SAR, China; 5 School of Nursing, The University of Hong Kong, Hong Kong, SAR, China; Kyoto University, JAPAN

## Abstract

Evidence-based practice and capacity-building approaches are essential for large-scale health promotion interventions. However, there are few models in the literature to guide and evaluate training of social service workers in community settings. This paper presents the development and evaluation of the “train-the-trainer” workshop (TTT) for the first large scale, community-based, family intervention projects, entitled “Happy Family Kitchen Project” (HFK) under the FAMILY project, a Hong Kong Jockey Club Initiative for a Harmonious Society. The workshop aimed to enhance social workers’ competence and performance in applying positive psychology constructs in their family interventions under HFK to improve family well-being of the community they served. The two-day TTT was developed and implemented by a multidisciplinary team in partnership with community agencies to 50 social workers (64% women). It focused on the enhancement of knowledge, attitude, and practice of five specific positive psychology themes, which were the basis for the subsequent development of the 23 family interventions for 1419 participants. Acceptability and applicability were enhanced by completing a needs assessment prior to the training. The TTT was evaluated by trainees’ reactions to the training content and design, changes in learners (trainees) and benefits to the service organizations. Focus group interviews to evaluate the workshop at three months after the training, and questionnaire survey at pre-training, immediately after, six months, one year and two years after training were conducted. There were statistically significant increases with large to moderate effect size in perceived knowledge, self-efficacy and practice after training, which sustained to 2-year follow-up. Furthermore, there were statistically significant improvements in family communication and well-being of the participants in the HFK interventions they implemented after training. This paper offers a practical example of development, implementation and model-based evaluation of training programs, which may be helpful to others seeking to develop such programs in diverse communities.

## Introduction

We describe the framework, development and results of a systematic evaluation of a 2-day training workshop for a community- and family-based positive psychology intervention project, entitled “Happy Family Kitchen Project” (HFK) in Hong Kong[1]. The training workshop and the HFK intervention program were delivered under the “FAMILY project, a Jockey Club Initiative for a Harmonious Society” (FAMILY project)[2]. We conducted a formal evaluation of the effectiveness of the training workshop at multiple time points to two years after the training.

Certain characteristics of our HFK were informed by the documented importance of community involvement to enhance acceptance of evidence-based interventions. Most evidence-based interventions were tested in academic settings, and were rarely sustained after the clinical trials that document their efficacy[3]. An important reason for this lack of translation is that the needs of community settings where most interventions were tested, were usually not taken into account during the development and implementation of the interventions[4]. Learning from the community-based participatory approach[5, 6], we gathered the cooperation, experience and input of social welfare organizations, schools and government departments to plan, implement and evaluate the HFK.

A second related demand came from concepts of prevention science. Effective, preventive interventions must be spread broadly and widely across the population, by individuals trained in practical and brief programs, and delivered to a large number of people at low cost [7]. The train-the-trainer educational model has been suggested as a mechanism to build workforce capacity[8]. Experts train the key stakeholders to deliver services[9], ideally in focused programs with minimum time and cost burden, resulting in efficient and effective utilization of available resources. In the social service sector, this train-the-trainer educational model has been implemented to train personnel who have served, among others, survivors of natural disasters [10–14], individuals with traumatic stress[15], victims with domestic violence[16] and ethnic minority citizens with health disparities[17]. It has also been adopted in preventive efforts such as perinatal depression screening[18] poison prevention[19], youth development support[20], parenting support[21], and health and mental care for the ageing education[22]. In addition, the train-the-trainer model has also been previously applied to increase social service workers’ interests in dissemination and implementation research in health[4].

Although there has been significant interest in the literature on the concept of the train-the-trainer program/workshop (TTT), identifying the gaps had guided the design of this study. First, few reports of TTTs evaluated the effectiveness of the training program[4, 14, 16, 18, 19, 21–26]. When TTTs in the social service were evaluated, it was done most typically immediately upon completion, or in the short term (for example, Polivka et al. 2006 examined training outcomes six weeks after training completion)[19]. As the effectiveness of an intervention depends partly on how well the interventionist to be trained is to conduct the program, and the goal of having a sustainable program that extends further than the immediate term, we designed our study to examine not only the gains maintained over two years, in addition to the outcomes of the interventions that the trainees delivered soon after training.

A second gap is that there has been no framework used to guide evaluation of these programs. The value of a model is to specify components that are important in training, and examine them separately to guide future improvements. The present training workshop reported in this paper was systematically evaluated in three aspects, including: (i) reactions to training content and design, (ii) changes in knowledge and attitude, and (iii) utilization, sharing and benefits to the service organization, adapted from the Integrated Model of Training Evaluation and Effectiveness (IMTEE)[27]. IMTEE was originally developed to assess training in the business world.

The training workshop, the intervention programs and the findings of HFK in this paper were under a citywide project entitled FAMILY project [2]. FAMILY Project was initiated and funded by The Hong Kong Jockey Club Charities Trust and conducted in collaboration with the School of Public Health of The University of Hong Kong (HKU-SPH). The aim of FAMILY project was to promote health, happiness, and harmony (3Hs) in Hong Kong families. Hong Kong is one of the most westernized and economically developed cities of China. As in much of the modernized world, traditional values that emphasize the importance of family are in danger of eroding. Long working hours and stressful urban lifestyles pose barriers for strong and supportive relationships among family members. Effective communication in a family is believed to enhance healthy family relationships[28, 29], which influence family well-being (family 3Hs)[2, 30].

Enhancement of protective factors that guard against the development of emotional and behavioural disorders is particularly important, especially in resource-poor areas where skilled mental health workers are a limited resource, and treatment is stigmatized. We selected the positive psychology framework as the basis for designing the community-wide interventions in HFK[1]. Positive psychology has been shown to be effective in the enhancement of psychosocial well-being [31]. A review by the originators of the framework [32], and a meta-analysis of 51 positive psychology interventions concluded that positive psychology intervention significantly enhanced well-being and decreased depressive symptoms[31]. Another recent review paper stated that positive psychology played an important role to be applied on setting guidelines for promoting work-family balance[33]. Interventions based on this framework can be widely applied in real-world settings[34]. Positive psychology also offers a health-enhancing rather than a disease prevention approach, consistent with the idea of supporting and developing psychological and family resources, while avoiding the stigma related to mental ill health. In our own work earlier in the FAMILY Project, we determined that the concepts were intuitively appealing to both social service sector staff and to members of the community[35].

Our training workshop aimed to meet the needs of social service workers from various participating social or community service organizations, and enhance trainees’ competence and performance in applying positive psychology constructs in designing and implementing their own community-based HFK intervention programs. The evaluation included qualitative focus group interviews at six months after training, and quantitative questionnaire assessments immediately before and after the delivery of the workshop, and again at six months, one year and two years after the workshop.

The primary objective was changes in learners (trainees), including competence (perceived knowledge, self-efficacy and attitude) and performance (practice) in conducting the HFK family interventions six months after the training. The secondary objectives were sustainability of the changes in competence and performance in conducting family intervention at longer term (1-year and 2-year follow up), and the effectiveness of the trainees’ implemented family interventions.

The current study makes some novel contributions to the literature in that: we formally evaluated the effectiveness of the training over a fairly long period, which is rare in the literature; and we utilized a framework to guide our evaluation that may be helpful to others who wish to evaluate their own training programs.

## Methods

### Ethical statement

Ethical approval was granted by Institutional Review Board (IRB) of the University of Hong Kong/Hospital Authority Hong Kong West Cluster. Written informed consents were obtained from the learners (trainees) and adult participants or parents of participants aged 18 years or below.

### Context of the train-the-trainer workshop

**Fig 1:**
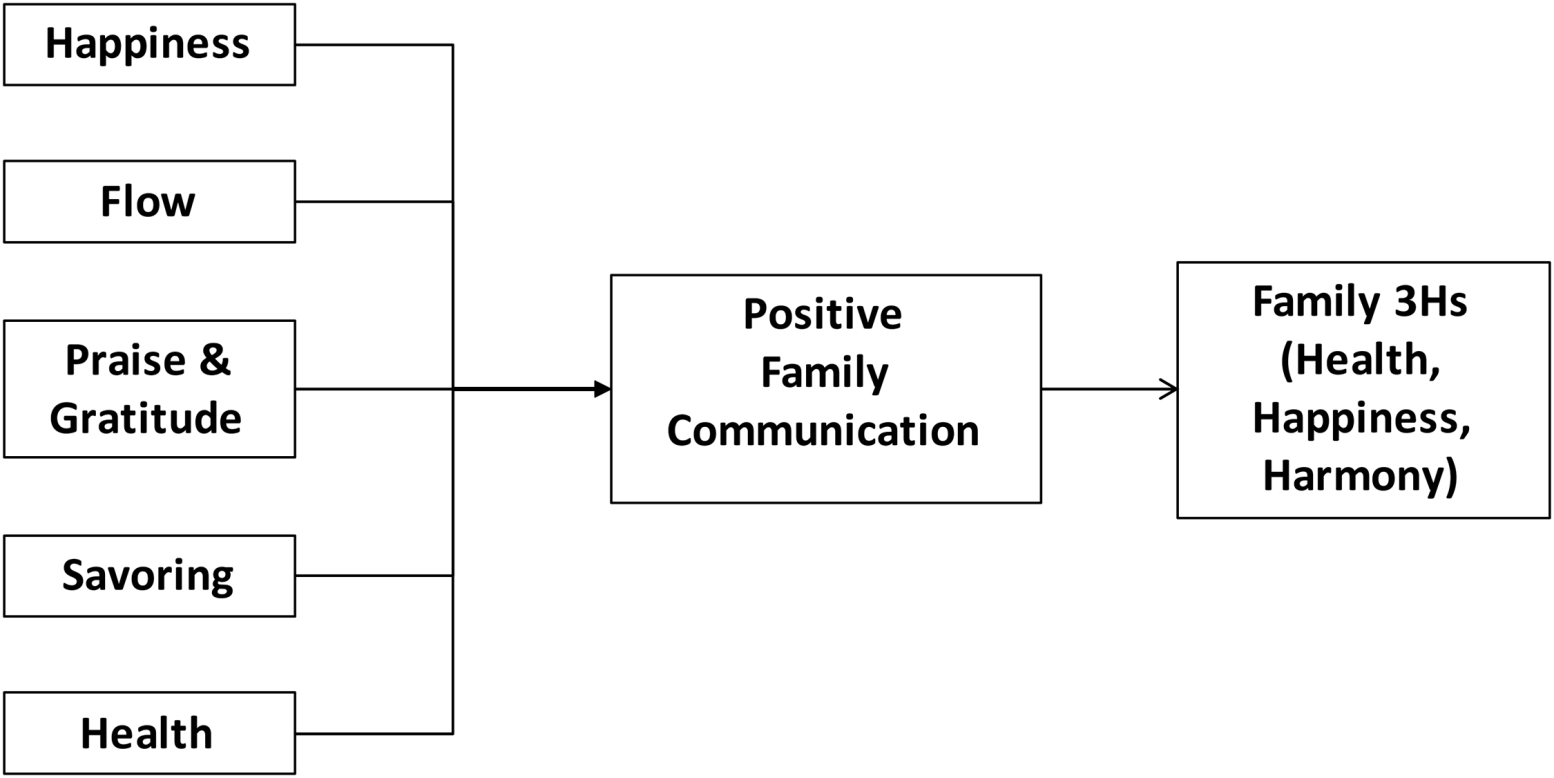
The conceptual framework of Happy Family Kitchen family intervention program.

The context of the current paper was the HFK in partnership with The Hong Kong Council of Social Service (HKCSS) (a non-government organization with 400 community organization members). The HFK was implemented in Yuen Long district, which was chosen because it was one of the most deprived districts in Hong Kong. This was the first large-scale community- and family-based intervention sub-project under the FAMILY project, preceded primarily by smaller scale, more intensive interventions[36]. The framework of HFK was based on promoting traditional Chinese values of cherishing family relationships[35]. We used the context of mealtimes that every Chinese recognizes an important time to connect with family members. Enjoying a meal together is the most important family reunion during festivities. The goal of HFK was to promote strong family relationships though the preparation and consumption of family meals. The five positive psychology themes were the basic units of the conceptual framework of the HFK intervention programs[32, 37–42] (Table 1) (Fig 1).

**Table 1:** Concepts and behavioural indicators of the five positive psychology themes.

Themes	Concept	Behavioural indicators
Happiness[37]	refers to a subjective sense of well-being (positive emotion and psychological state), as well as the sense that life is worthwhile	-share happy experiences with family over mealtime
		-wait for everyone to be seated before starting to eat, and say “Let’s eat together!” towards each other
		-reminisce one’s experience that makes you or your family happy every day
Praise and Gratitude [38, 41]	is a combination of the expression of thankfulness and an emotional sense of appreciation	-praise the strength and goodness of your family members
		-express gratitude to family members by words
		-express gratitude to family members by actions, e.g. massage, serving tea
Flow [39]	is a personal experience with greatest satisfaction when people were totally immersed in and concentrating on what they are doing	-prepare/ clear/ wash dishes, etc. together with family members
		-look for family members’ character strengths during cooking and dining
		-focus on cooking/ dining with family, without doing anything else
Savoring [42–44]	is paying attention to the current source of pleasure, and consciously enjoying feelings as they unfold	-slow down the pace of eating
		-savour food by observation, focus on its ‘colour’, ‘smell’ and ‘taste’, treasure good time
		-treasure good time when dine with family, e.g. stay with family on table though finish dining
Health [40]	focuses on the development of positive thought (optimism), improving resilience, and nutrition	-cook/ choose food with the principal of low fat, low sodium, low sugar and high fibre
		-write down encouraging words and post them up at home to let yourself and your family members see them
		-say supportive words to your family members

The intervention that this TTT prepared to design focused on communicating the value of one of the five positive psychology themes (‘Praise and Gratitude’, ‘Flow’, ‘Happiness’, ‘Health’, and ‘Savoring’), within the context of food preparation and dining at home. The trainees were exposed to the concept and given a chance to discuss its value with other group members. This was followed by activities that elicited the concept. Homework was assigned in the form of theme-eliciting activities. For example, in the ‘Praise and Gratitude’ theme, participants were asked to keep a diary of events that made them feel grateful for a family member, and how they shared some of these events during a meal with family members. Each community-based family intervention program consisted of two core intervention sessions and one booster session. The two core sessions targeted on the positive family communication methods and skills, which were run in the form of group activities and homework assignment. The booster session was held to consolidate the information of the core sessions to allow the participants to share their experience. The training and intervention contents were determined by the multidisciplinary research team as relevant and easily grasped by the Hong Kong population. The research team included academic public health professionals, a nurse, a clinical psychologist, a dietitian, other academic staffs with experience in developing and implementing intervention programs in the community, and social service stakeholders, who also designed and conducted the training. We hypothesized that the longer time spent with family, and better communication skills would promote family well-being (Family 3Hs). Our ultimate outcome was family well-being, defined as family health, harmony and happiness[2, 30](Fig 1).

The HFK steering committee included academics from HKU-SPH and community agency representatives from HKCSS who directed the training program, approved the proposals of intervention programs (designed by the trainees) from the participating social service organizations, and distributed the funding (about HK$10,000, ie US$ 1300) for each program before the programs were implemented. Staff from the organizations that expressed an interest in participating in HFK (a maximum of three persons from each service unit) attended the TTT. They would serve as the interventionists, who were responsible for the development and implementation of the family intervention programs with the same overall objectives of HFK. After completing the TTT, the trained interventionists selected and incorporated one of the five themes of positive psychology that they believed would be best for the participants of their intervention programs. The design of the training allowed the interventionists the maximum amount of flexibility using various activities to improve family communication in their service setting. The trainees were required to submit their program plans (a run-down that included information about activities developed according to the theme) to the HFK. The committee checked whether the programs fit the set criteria of approving funding to the participating social service units, and suggested improvements to be made before resubmission. The criteria included that: a) the trainees select a single positive psychology theme for their intervention; b) the activities described were consistent with this theme and were based on family interactions; c) the intervention was organized as two core intervention sessions and one booster session, with at least one-hour intervention per session; d) the intervention had an explicit structure that included an introduction, a series of activities, and assessment; e) the allocation of staff was consistent with the demands of the activities as designed. The program approval step was designed to ensure that the themes were appropriately incorporated and could meet the aims of HFK. After approval, the trainees implemented the family interventions, and assisted the HKU-SPH research team in the follow-up evaluation of their service targets at each time-point. At each intervention session, an observer from the research team completed a checklist that corresponded to the approved plans to ensure the fidelity of the interventions.

#### Pre-training phase

A needs assessment was conducted by focus group interviews with social workers on 24 September 2010. This assessment aimed: (i) to identify the needs, resources and feasibility of developing and evaluating a family intervention program; and (ii) to explore ideas from social workers and enhance the acceptability and applicability of the TTT. This information was important to frame the training objectives and guided the design and content of the training workshop and practice manual.

#### Training phase

We embedded the positive psychology constructs in a variety of activities to suit the broad range of educational background and age of participants who were served by the social service organizations. The training workshop consisted of four sessions (three hours per session) held over two days on 19 and 23 November 2010. Table 2 shows the details of the curriculum. We introduced the overall project aims and the conceptual framework, including the general construct of positive psychology, five specific positive psychology themes, and the concept of family well-being. We also introduced the concepts of evidence-based, evidence-generating research and evaluation methods. We discussed program planning and proposal writing (necessary to obtain funding from the granting agency for an individual program). In addition, the importance of the quality and fidelity of the intervention delivered, and the acceptability and feasibility of the program, were described with an emphasis on how to identify areas for improvement. The concept of process evaluation, which included six components of fidelity, dose delivered, dose received, reach, recruitment and context[45], was introduced. Process evaluation was used to monitor and document program implementation and could aid in understanding the relationship between specific program elements and program outcomes[45].

**Table 2:** The curriculum of train-the-trainer workshop.

Day 1	Day 2
***Session 1***	***Session 3***
To introduce the key components of the project	To introduce specific positive psychology themes: ‘Savoring’ and ‘Health’ with experiential activities
-Overall project aims	
-Conceptual framework	
-The general construct of positive psychology	
-Family well-being (Family happiness, health, harmonious)	
-Community-based participatory approach	
To introduce specific positive psychology theme: ‘Happiness’ with experiential activities	
***Session 2***	***Session 4***
To introduce specific positive psychology themes: ‘Praise and Gratitude’ and ‘Flow’ with experiential activities	To introduce knowledge on healthy eating, choice of food, and the relationship between food and emotion
To introduce the key components of research study	To demonstrate how to practice the healthy eating and its cooking methods.
-Evidence-generating research method	
-Evaluation framework: methods and time frame	To provide guidance on program planning and proposal writing
-The six domains of process evaluation: ‘Fidelity’^1^, ‘Dose delivered’^2^, ‘Dose received’^3^, ‘Reach’^4^, “Recruitment’^5^ and ‘Content’^6^	

Notes:

^1’^Fidelity’ refers to the extent to which intervention was implemented as planned;

^2^ ‘Dose delivered’ refers to the amount or number of intended units of each intervention or component delivered or provided by interventionists;

^3^ ‘Dose received’ refers to (a) the exposure (the extents to which participants actively engaged with, interacted with interventionists and other participants, were receptive to, and/or used materials or recommended resources) and (b) satisfaction (participants’ satisfaction with program and interactions with staff and others).

^4 ‘^Reach’ refers to the proportion of the intended priority audience that participated in the intervention;

^5 ‘^Recruitment’ refers to the procedures used to approach and attract participants;

^6 ‘^Context’ refers to the environmental issues that might affect the intervention implementation or study outcomes.

In communicating the content, diversified learning methods were used. The trainees not only received some didactic instruction, but also were engaged in games, role plays and group discussions. The emphasis was on experiential learning. We used different methods to engage actively the trainees to act as the expected participants of their proposed family interventions, including games and role play. We merged positive psychology themes into group activities and gave trainees’ hands-on experience that they might engage and communicate the messages in large groups. A practice manual (first version) was prepared by the research team to serve as a practical guide for the design and implementation of the family intervention programs and was distributed to the trainees. The manual covered the concepts of five positive psychology themes, the behavioral indicators of the five themes and suggested family intervention activities for each theme (Table 1) to reinforce the teaching and learning imparted in the workshop.

#### Post-training phase

In the post-training phase, we evaluated: (i) changes in competence and performance, which were knowledge, attitude and practice of the trainees; (ii) quality of their implemented family-intervention programs; and (iii) changes in the key outcomes of participants’ family well-being, after joining the interventions developed by the trainees. Two focus group interviews were conducted in May 2011 after the trainees had implemented their family interventions, approximately six months after training. The interview focused on trainees’ reactions towards the training workshop, and the issues/difficulties faced when implementing the family-intervention programs. We explored the overall design, barriers in implementation and effectiveness of the workshop, and investigated the usefulness and applicability of the training content and practice manual. On 7 July 2011, at the completion of HFK project, a refined version of the practice manual (108 pages) was developed and was widely disseminated to other social service workers and community stakeholders in Hong Kong. In addition, the results and experiences of HFK were shared through a Practice Wisdom Forum to influence the attitude and practice of social service stakeholders.

### Evaluation of outcomes: Study measures

#### Reactions to training content

We asked the trainees to grade the content, quality, level of utility and overall performance of the training program, and indicate how difficult it was to apply the training content in program design and implementation. Responses were made on a five-point Likert scale, ranging from “1 = improvement needed” to “5 = very good” in the performance of the training program, and from “1 = very difficult” to “5 = very easy” in the application of training content. A higher score indicated a more favorable response.

#### Changes in knowledge and attitude

We assessed the “changes in knowledge and attitude” component of the IMTEE model, by asking trainees to indicate the extent of agreement with statements about three domains: (i) cognitive learning (perceived knowledge) in relation to the general constructs of positive psychology (three items; for example, “I know the key components of positive psychology.”); (ii) self-efficacy (confident I have the skills) to apply the general construct of positive psychology in program design (three items; for example, “I am confident I can apply the concept and techniques of positive psychology in designing my program.”); and (iii) attitudes towards the practice of positive psychology (three items; for example, “Positive psychology is effective at enhancing family well-being.”). Responses were made on a six-point Likert scale, ranging from “1 = strong disagree” to “6 = strongly disagree.” A higher score indicated a better performance.

#### Using and sharing positive psychology construct and benefits to the service organization

We asked how often the trainees utilized positive psychology constructs in practice (six items; for example “In the past six months, how often did you bring out the concept of positive psychology in activity design?”). Responses were made on a five-point Likert scale, ranging from “1 = never” to “5 = always.” Questions on improvement on activity design and developing family activities or policies were asked (two items). Responses were made on a five-point Likert scale, ranging from “1 = no improvement at all” to “5 = much improved.” We also asked whether they plan to apply the training content into their future program design and share the knowledge and skills learnt with others (two items) Response options were “yes”, “no”, and “not sure.” The questionnaire appears in S1 Appendix.

We evaluated the impact of the programs delivered by trainees after the training was completed, to their participants. We used a measure of family well-being created for the FAMILY project, which asked the service targets to indicate their assessment of their family harmony, happiness and health by three questions: “Do you think your family is harmonious?”; “Do you think your family is happy?” and “Do you think your family is healthy?”. Each item allowed response on a 0 to 10 scales ranging from “not at all harmonious / happy / healthy” to “very harmonious / happy / healthy.” A higher score indicated a more positive perception of family well-being. The internal consistency (Cronbach’s Alpha) was 0.89.

### Statistical analysis

Analyses were conducted using SPSS version 20.0. All significance tests were 2-sided with a 5% level of significance. The principle of intention-to-treat analysis was adopted. Missing data of trainees who resigned from the participating organizations, were lost to follow up, or declined to complete the questionnaire, were replaced by their baseline values. Repeated-measures Analysis of Variance was employed to compare the means at five time-points. Sensitivity analysis was performed by using per protocol analysis, which included those trainees with assessments at 2-year follow up and excluded those with missing data. All qualitative interviews were audio-taped and transcribed verbatim in Cantonese in order to capture every nuance of expression unique to the dialect. At least 10% of the transcripts were double-checked with the recordings. Coding was processed by two project team members, one of whom attended the interviews. This arrangement ensured objectivity during analysis. Transcripts were analysed by thematic content analysis, following the guidelines recommended by Morse and Field[46]. Each transcript was analysed sentence by sentence and coded for the respondents’ meaning. The transcripts were reviewed again by another member of the project team to validate the thematic analysis and to ensure that all meaningful interview data had been analysed.

## Results

**Fig 2:**
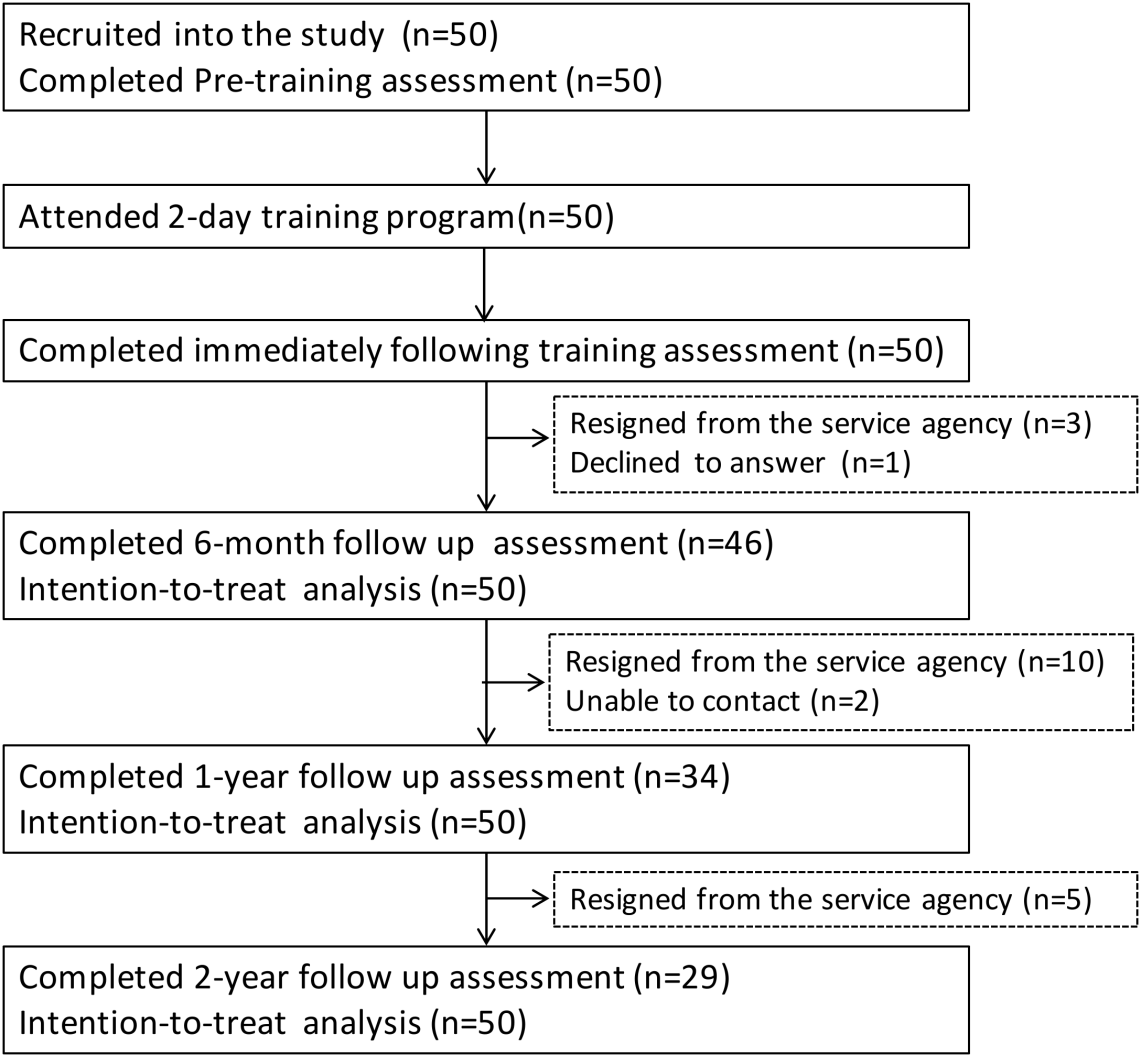
The trainees' disposition.

Of the 50 social workers, who were assigned by their participating service units and attended the training workshop, three trainees resigned later from their service units, and one trainee declined to complete the questionnaire at the 6-month assessment. Eleven trainees resigned from the service units, and two could not be contacted at one-year follow up. At 2 years, five more trainees resigned. Fifty questionnaires at pre-training (T1), 50 immediately following training (T2), 46 at 6-month (T3), 34 at 1-year (T4) and 29 at 2-year follow up (T5) were thus collected (Fig 2). The internal consistency (Cronbach’s Alpha, α) of each domain of the questionnaire in related to the general concept of positive psychology are: perceived knowledge, α = 0.94; self-efficacy, α = 0.95; attitude towards the practice, α = 0.90; and application, α = 0.96.

Twenty trainees, representing 18 social service units joined the focus group interviews, after the implementation of their first family intervention, approximately three months after training. Table 3 shows the trainees’ characteristics. There were no significant differences in the demographic characteristics of trainees who participated in the focus group and those who did not.

**Table 3:** Demographic characteristic of all trainees (n = 50) and those in focus group (n = 20).

	All (n = 50)	Focus group (n = 20)
	Number (%)	Number (%)
Age group, years		
18–24	5 (10)	2 (10)
25–34	21 (42)	8 (40)
35–44	11 (22)	4 (20)
> = 45	13 (26)	6 (30)
Female	31 (62)	12 (60)
Tertiary degree or above	36 (72)	15 (75)
Registered social worker	41(82)	19 (95)
Social service experience, years		
<1	6 (12)	3 (15)
1–4	14 (28)	6 (30)
5–9	9 (18)	2 (10)
10–19	18 (36)	8 (40)
≥ 20	3 (6)	1 (5)
Service targets		
Family	29 (58)	13 (65)
Children	23 (46)	7 (35)
Teenagers	18 (36)	7 (35)
Had previous exposure to positive psychology	22(44)	12 (60)

### Reactions to training content

Immediately following training, the majority (94%) of trainees rated the overall training “good” or “very good”. Over 87% of the trainees rated the content, quality and level of utility as “good” and “very good”.

At focus group interview, the trainees commented that the training was well-organized. The concepts of research design and the goal of the intervention programs were clearly delivered in the training program.

*“(We) had the key concepts*, *so we carried out*…*the program under this (framework)*…, *which is very important*. *(The training) It helped us with designing and implementing the program*. *We could design (the program) with the key messages of what we’ve learnt in the workshop*, *and the training helped us get solid direction quickly*.*”*

*“(We) could tailor the design according to our service targets*, *which is important for us as we have many different constituents*. *I have gained in-depth experience from this (training workshop)*. *In fact*, *we thank you for the well-prepared training workshop which has been very helpful to us”*

The Practice Manual was very useful in developing the intervention programs, and the experiential learning method was much appreciated.

*“(The workshop) was really useful*! *When I tried to rely on recall alone*, *many of the details were forgotten*, *so the practice manual was great and useful*! *I read it over and over again*.*”*

*“I think the method was very helpful*, *for example*, *role play*. *(The training) It treated us like a participant (the clients of our program)… I think it’s good (for us) to try (those games)*…*”*

The application of positive psychology in the intervention program was simple and engaging to their service targets.

*“It was easy to deliver (the message) to them (the clients of our programs)*. *Positive Psychology is actually not that complicated*. *They (the clients of our programs) could take (the message) home*!*”*

### Changes in knowledge and attitude

Table 4 shows a significant increase in perceived knowledge in relation to positive psychology with a large effect size immediately following the training (Cohen’s d = 1.09, p < 0.001). These effects were sustained at six months (Cohen’s d = 0.80, p < 0.001), one year (Cohen’s d = 0.58, p < 0.001), and two years (Cohen’s d = 0.37, p<0.001). Self-efficacy for applying positive psychology constructs in program design significantly increased with a large effect size (Cohen’s d = 1.15, p < 0.001) immediately following the training and was sustained at six months (Cohen’s d = 0.80, p < 0.001), one year (Cohen’s d = 0.68, p<0.001) and two years (Cohen’s d = 0.53, p<0.001). There was no significant increase in the attitude towards the practice of positive psychology immediately following the training (Cohen’s d = 0.19, NS). However, a significant increase appeared at six months (Cohen’s d = 0.46, p = 0.011), though not at one year and two years. The declines in effect size at one and two years were mainly due to the replacement of missing data by baseline values. Per-protocol analysis showed similar findings but with greater effect size at one and two years (Table 5).

**Table 4:** Trainees’ knowledge, attitude and application in relation to positive psychology over time: intention-to-treat analysis (n = 50).

						Difference between			
	Pre-training	Immediately after the training	6 months	1 year	2 years	Pre-training and immediately after training	Pre-training and 6 months	Pre-training and 1 year	Pre-training and 2 years
			Mean score ± SD				Cohen’s d^c^	/ p-value^d^	
Perceived knowledge of the general concept of positive psychology^a^**	3.5 ± 1.0	4.4 ± 0.6	4.2 ± 0.8	4.0 ± 0.9	3.8 ± 1.0	1.09 / <0.001	0.80 / <0.001	0.58 / <0.001	0.37 / <0.001
Self-efficacy in relation to using positive psychology constructs to design interventions ^a^**	3.2 ± 1.0	4.1 ± 0.6	3.9 ± 0.8	3.9 ± 1.0	3.8 ± 1.0	1.15 / <0.001	0.80 / <0.001	0.68 / <0.001	0.53 / <0.001
Attitude towards the practice of positive psychology ^a^	4.3 ± 0.6	4.4 ± 0.7	4.6 ± 0.7	4.4 ± 0.5	4.4 ± 0.6	0.19 / 0.261	0.46 / 0.011	0.23 / 0.226	0.21 / 0.226
Application of positive psychology in interventions^b^**	2.1 ± 1.0	-------	3.2 ± 1.0	3.1 ± 0.5	2.6 ± 0.9	-------	1.22 / <0.001	1.0 / <0.001	0.56 / <0.001

Repeated-measure was employed to compare the mean at five time-points:

** p value <0.001

Factor scores were presented: perceived knowledge (3 items), self-efficacy (3 items), attitude towards the practice (3 items), and application (6 items)

^a^ 6-point Likert scale: 1 = strongly disagree; 2 = disagree; 3 = slightly disagree; 4 = slightly agree; 5 = agree; 6 = strongly agree

^b^ 5-point Likert scale: 1 = never; 2 = rarely; 3 = sometimes; 4 = often; 5 = always

^c^ Effect size (Cohen’s d): small = 0.20, medium = 0.50 and large = 0.80

^d^ p value for the difference between two time-points

**Table 5:** Trainees’ knowledge, attitude and application in relation to positive psychology over time: per-protocol analysis (n = 29).

						Difference between			
	Pre-training	Immediately after the training	6 months	1 year	2 years	Pre-training and immediately after training	Pre-training and 6 months	Pre-training and 1 year	Pre-training and 2 years
			Mean score ± SD				Cohen’s d^c^/	p-value^d^	
Perceived knowledge of the general concept of positive psychology^a^**	3.5 ± 1.0	4.4 ± 0.7	4.3 ± 0.7	4.4 ± 0.5	4.2 ± 0.9	1.00 / 0.001	0.97 / <0.001	1.08 / <0.001	0.67 / 0.005
Self-efficacy in relation to using positive psychology constructs to design interventions ^a^**	3.2 ± 0.8	4.2 ± 0.5	4.2 ± 0.6	4.3 ± 0.5	4.1 ± 0.8	1.36 / <0.001	1.34 / <0.001	1.50 / <0.001	1.02 / <0.001
Attitude towards the practice of positive psychology ^a^*	4.4 ± 0.6	4.5 ± 0.5	4.8 ± 0.7	4.6 ± 0.6	4.6 ± 0.6	0.32 / 0.151	0.68 / 0.009	0.36 / 0.197	0.36 / 0.162
Application of positive psychology in interventions^b^**	2.2 ± 1.1	-------	3.5 ± 0.9	3.5 ± 0.7	3.1 ± 0.6	-------	1.41 / <0.001	1.54 / <0.001	1.16 / <0.001

Repeated-measure was employed to compare the mean at five time-points:

** p value <0.001

* p value <0.001

Factor scores were presented: perceived knowledge (3 items), self-efficacy (3 items), attitude towards the practice (3 items), and application (6 items)

^a^ 6-point Likert scale: 1 = strongly disagree; 2 = disagree; 3 = slightly disagree; 4 = slightly agree; 5 = agree; 6 = strongly agree

^b^ 5-point Likert scale: 1 = never; 2 = rarely; 3 = sometimes; 4 = often; 5 = always

^c^ Effect size (Cohen’s d): small = 0.20, medium = 0.50 and large = 0.80

^d^ p value for the difference between two time-points

### Using and sharing positive psychology constructs

Compared to baseline, the trainees were more likely to apply positive psychology in designing activities to improve family health, happiness and health (Cohen’s d = 1.22, p <0.001) at six months. This increase was sustained at one year (Cohen’s d = 1.0, p < 0.001) and two years (Cohen’s d = 0.56, p < 0.001) (Table 4). At six-month assessment, about half of the trainees reported improvements in program design (n = 27, 54%) and developing family activities/policy for their organization (n = 25, 48%), after learning the five specific positive psychology themes. Forty-four (88%) trainees indicated that they would apply the positive psychology constructs they had learned into their activities in the future. Thirty-eight (78%) trainees stated that they would share the knowledge and skills learnt with other colleagues or organizations.

### Results of family intervention programs by trainees

Eventually, all 23 proposals were funded. The trainees successfully designed and delivered 23 community-based family-intervention programs from January to June 2011. Six hundred and twelve families with 1419 participants joined the HFK family-intervention programs and completed the assessments. The research protocol and details of the result had been reported in a separate paper and mentioned in a HFK evaluation report [1, 47]. There was a significant increase in family communication time at six weeks and communication score at three months, compared to baseline. In addition, there were significant improvements in all measures of family well-being (family health, happiness and harmony) at six weeks, but only the effect on family happiness sustained at three months.

## Discussion

The 2-day TTT enhanced the competence and performance of the trainees with large effect size. These results were consistent from immediately following the training, to two years after training. The trainees highly appreciated the workshop and its teaching and learning strategies, especially the experiential learning method and the practice manual.

We demonstrated a successful community-academic research partnership and development of a capacity-building workshop for social service workers. The TTT educational model ensured diffusion of innovation[48]. It built confidence and cultivated the application of theory-based interventions of social service workers. Social service sector workers act as a link between academic researchers and communities[49]. This workshop laid a good foundation for deeper collaboration between community-based social service organizations and academics in the future.

Prior to developing this TTT, we familiarized ourselves with adult learning approaches. A recent review suggested that blended teaching and learning approaches effectively disseminated and implemented curricula to social and health professionals in TTT[9]. The practice manual provided key information and instructions to the trainees, which enhanced their confidence in designing and implementing the activities[50]. We adopted the experiential learning approach, and interactive teaching strategies, including games, sharing sessions and group discussions. These strategies also should be better than didactic programs in managing the challenge of rapid engagement and also immersion in the concept that the interventionist hopes to communicate. These strategies also allow “planning”, and an important component in many behavior change models[51] through practice, and the opportunity to receive immediate feedback. Experiential learning approaches have been noted to be powerful teaching and learning tools, and to promote professional growth of social workers[20, 52]. The qualitative comments on this study also suggested that these strategies could engage and teach effectively. Furthermore, social service workers were trained to design and deliver a broad range of programs, not a specific, restricted and top down manualized intervention. The utilization of these strategies offered a model of what they might emulate in their own run-downs.

The TTT was assessed systematically by a model-based evaluation[27], including the entire process of engagement, capacity building and intervention. There were 21 trainees (42%), who were treated as lost to follow up because they resigned from the servicing agencies, were transferred to other social service units, or refused to answer the questionnaires at 2-year assessment (Fig 2). The main results (reactions to the training content, changes in knowledge and attitude, and using and sharing of positive psychology constructs) were consistent with the focus in the training on the acceptability and applicability of the content to trainees’ needs, the enhancement of trainees’ competence and performance, and the results of the implemented family intervention. The effect was sustained up to two years after the training. The sustained effects might be explained by the use of strategies that promoted practices during the training, and a comprehensive manual. Experiential teaching and learning methods can build concrete experience, observation and reflection, and promote active implementation[52]. The comprehensive practice manual might have acted as a “booster” by allowing repeated references back to the training material, and strengthened trainees’ confidence in their ability to design and conduct their programs. However, the intervention effects diminished over time, which might be explained by the diversion to other activities that when the stimulus for change is gone[3], and the assumption of no improvement in learning and practice after training on those trainees, who were lost to follow up, or declined to complete the questionnaire. The principle of intention-to-treat analysis was adopted, which included every trainee in the group, regardless of subsequent deviation from the protocol [53]. It avoided overoptimistic estimates of the efficacy of the training workshop resulting from the removal of non-compliers [54]. We also analysed the data with the last observed values carry-forward to replace the missing data, and the per-protocol analysis. These results showed persistent effect size to two years.

Our TTT presented differs in important ways from most of the TTTs reported in the literature. Most of the TTTs in the literature were 3-day[10, 13, 14, 20, 24], 5-day[4], 8-day[55] or 10-day[25] training programs. Our study cannot assess whether this shorter TTT was as effective as a longer one. However, from a practical point of view, brief programs require fewer resources to develop and disseminate. We are able to demonstrate that even shorter programs can be effective, and this may be important information, which is an important consideration in low resource areas. Our study improves on the current literature by including formal evaluation of the training program, using both qualitative and quantitative assessment methods, and measuring effects up to two years following the training. Many reports of TTTs presented only descriptive information such as the theoretical framework, development, and design [10, 11, 13, 15]. Some studies utilized the qualitative evaluation such as process evaluation [55] and narrative feedback[20]. Quantitative evaluations of the effectiveness of TTT programs were rarely reported. Where formal evaluation was included, investigators compared changes from before to immediately after the training[16, 21, 22, 24, 26] or reported post assessment results immediately after the training but without a pre-test[14, 18, 23, 25, 56]. A rare example of follow-up evaluation was reported by the “Be Poison Smart” programs, which showed an increase in knowledge scores only of the participants at six weeks after training.

Our TTT had high acceptability and applicability of the training content and design. There were significant improvements in knowledge, self-efficacy and attitude related to the positive psychology constructs and program design and implementation. Researchers suggested that knowledge, skills and attitudes were powerful determinants of quality of work, and influenced performance subconsciously[57]. Those factors could explain the performance of the trainees as demonstrated by the effectiveness of their implemented family intervention programs[58].

Several suggestions might be derived from our findings for developing similar training programs. The utilization of an experiential learning approach, or “learning by doing” and interactive teaching strategies, may be particularly beneficial for engaging and retaining the interest of individuals from a broad set of educational and social backgrounds, who have minimal experience with absorbing information through didactic teaching. Second, investigators may want to consider the value of teaching not just a specific manualized intervention, but including a framework such as the Logic Model. By empowering social service workers to utilize some simple tools to design and evaluate their own future programs, the positive effects of the academic-community partnership can extend well beyond the life of a single trial. Finally, most training programs delivered as a part of developing trials, which was not formally evaluated. We propose that adding an evaluation component to training programs may shed light on why some interventions implemented in the field are successful or not.

There were several limitations in our study. First, because validated questionnaires were not available, we developed our outcome-based questionnaire to assess the changes in trainees. We measured perceptions and not actual knowledge and skills. Perceived knowledge and skills, may not reflect actual knowledge and skills acquired and can be influenced by the individual’s personality, self-perception[59], and may be under- or over-estimated depending upon numerous factors in play at the time when completing the questionnaire. In addition, social desirability bias might have exaggerated the positive findings. We did get indirect information about actual knowledge and skills by examining the trainees’ proposals, the design and content of the programs and the delivery process, which fulfilled our requirement. Furthermore, the programs implemented by the trainees had high-fidelity assessment results and were effective even though they were brief, different in content (but similar objectives and evaluation) and delivered in the community. Objective measures or tests/examinations of specific knowledge and skills, and a control group of trainees who do not receive the program would provide stronger evidence in future studies. Our study had a high attrition rate and the small sample size. We have noted that retention was closely related to the stability of the interventionists who formed relationships with the participants. Some collaborating service organizations had high turnover rates over the two-year study period (36%, n = 18). Our sample size was small, and the workers we included might not be representative of their profession across other agencies. Replications with more workers and in other contexts are needed. We cannot be certain about which of our strategies resulted in effective learning. Elucidation of the active ingredients of an intervention and the mediation components that are responsible for change, typically are undertaken after an intervention is proven to be effective. Examination of the effective components of our training strategies would be a future direction for research, and may allow refinement of the program for broader dissemination. Using a control group would be desirable. Finally, the TTT was delivered to empower the trainees to design and implement specific programs; the pledge of the funding support if their proposals were approved could be a strong incentive for their learning and practice.

To conclude, the training workshop was preventive, relatively brief, with quantitative and qualitative evaluations, and follow up, that included not only the effects on the trainees two years after the program, but also the positive effects on the participants of the interventions they designed and delivered. TTTs in public health may offer a model for cost-effective training and interventions to benefit many social and health service workers and large numbers of service targets. Evidence from this study is the first step towards the rational development of training programs that promote sound social service practice utilizing existing resources effectively.

(5804 words)

## Supporting Information

S1 Appendix Questionnaire for the train-the-trainer workshop. (DOCX)
