# Supplementary material for: Development and Two-Year Follow-Up Evaluation of a Training Workshop for the Large Preventive Positive Psychology Happy Family Kitchen Project in Hong Kong
Source: PLoS One. 2016 Jan 25;11(1):e0147712. doi: 10.1371/journal.pone.0147712 (PMC4726595; doi:10.1371/journal.pone.0147712)
Supplement: S1 Appendix — (DOCX) [file pone.0147712.s001.docx]

**Appendix 1 Questionnaire for the train-the-trainer workshop of Happy Family Kitchen Project**

| Please select to what extent you agree with the following sentences about positive psychology  **Part 1: Perceived knowledge of the general concept of positive psychology** | | | | | | | | | | | | | | | | |
| --- | --- | --- | --- | --- | --- | --- | --- | --- | --- | --- | --- | --- | --- | --- | --- | --- |
|  | | **Strongly disagree** | | | **Disagree** | | | **Slightly disagree** | | **Slightly agree** | | **Agree** | | | **Strongly Agree** | |
| 1.1 I havekknowledge of positive psychology. | | ➀ | | | ➁ | | | ➂ | | ➃ | | ➄ | | | ➅ | |
| 1.2 I have a basic awareness of the mechanics of positive psychology. | | ➀ | | | ➁ | | | ➂ | | ➃ | | ➄ | | | ➅ | |
| 1.3 I know what the key components of positive psychology are. | | ➀ | | | ➁ | | | ➂ | | ➃ | | ➄ | | | ➅ | |
| **Part 2: Self-efficacy in relation to using positive psychology constructs to design interventions** | | | | | | | | | | | | | | | | |
|  | | **Strongly disagree** | | | **Disagree** | | | **Slightly disagree** | | **Slightly agree** | | **Agree** | | | **Strongly Agree** | |
| 2.1 I can master the techniques of positive psychology. | | ➀ | | | ➁ | | | ➂ | | ➃ | | ➄ | | | ➅ | |
| 2.2 I can apply the techniques of positive psychology. | | ➀ | | | ➁ | | | ➂ | | ➃ | | ➄ | | | ➅ | |
| 2.3 I know how to bring out the concept of positive psychology in program design. | | ➀ | | | ➁ | | | ➂ | | ➃ | | ➄ | | | ➅ | |
| **Part 3: Attitude towards the practice of positive psychology** | | | | | | | | | | | | | | | | |
|  | | **Strongly disagree** | | | **Disagree** | | | **Slightly disagree** | | **Slightly agree** | | **Agree** | | | **Strongly Agree** | |
| 3.1 Positive psychology can provide direction to program planning. | | ➀ | | | ➁ | | | ➂ | | ➃ | | ➄ | | | ➅ | |
| 3.2 Positive psychology is a worthwhile practice. | | ➀ | | | ➁ | | | ➂ | | ➃ | | ➄ | | | ➅ | |
| 3.3 Positive psychology is an ideal way to guide to family health, happiness and harmony. | | ➀ | | | ➁ | | | ➂ | | ➃ | | ➄ | | | ➅ | |
| **Part 4: Application of positive psychology in interventions** | | | | | | | | | | | | | | | | |
| ***In the past 6 months, how often did you:*** | | | | **Never** | | | **Rarely** | | **Sometimes** | | | | **Often** | | | **Always** |
| 4.1 Bring out the concept of positive psychology in program design. | | | | ➀ | | | ➁ | | ➂ | | | | ➃ | | | ➄ |
| 4.2 Involve the 5 principles of positive communication in program design. | | | | ➀ | | | ➁ | | ➂ | | | | ➃ | | | ➄ |
| ***In the past 6 months, how often did you encourage the program participants to do the following:*** | | | | **Never** | | | **Rarely** | | **Sometimes** | | | | **Often** | | | **Always** |
| 4.3 Link the concept of positive psychology to family relationship. | | | | ➀ | | | ➁ | | ➂ | | | | ➃ | | | ➄ |
| 4.4 Apply positive psychology to improve family health. | | | | ➀ | | | ➁ | | ➂ | | | | ➃ | | | ➄ |
| 4.5 Apply positive psychology to improve family happiness. | | | | ➀ | | | ➁ | | ➂ | | | | ➃ | | | ➄ |
| 4.6 Apply positive psychology to improve family harmony | | | | ➀ | | | ➁ | | ➂ | | | | ➃ | | | ➄ |
| **Part 5: Sharing positive psychology constructs and benefits to the service organization beyond the specific intervention designed** | | | | | | | | | | | | | | | | |
|  | **No improvement at all** | | **No improvement** | | | **Basically did not improve** | | | | | **Improved** | | | **Much improved** | | |
| 5.1 Did you improve your broad knowledge and skills in designing and implementing activity within your interventions after this HFK training workshop? | ➀ | | ➁ | | | ➂ | | | | | ➃ | | | ➄ | | |
| 5.2 Did you improve your broad knowledge and skills on policy development within your organization after this HFK training workshop? | ➀ | | ➁ | | | ➂ | | | | | ➃ | | | ➄ | | |
|  | | | | | | **Yes** | | | | | **No** | | | **Not sure** | | |
| 5.3 In the future, do you plan to apply any of the five themes of positive communication in the activity design of the activities beyond this project? | | | | | | ➀ | | | | | ➁ | | | ➂ | | |
| 5.4 In the future, do you plan to share the knowledge and skill learnt in this training with other colleagues or organizations? | | | | | | ➀ | | | | | ➁ | | | ➂ | | |
